# Diagnostic methods and written advice for acute otitis media in primary health care

**DOI:** 10.1080/02813432.2024.2352444

**Published:** 2024-05-15

**Authors:** Veronica Frey Esgård, Ida Lindman, Anja Maria Braend, Guro Haugen Fossum, Thorbjörn Lundberg, Anna Moberg, Lena Nordeman, Chrysoula Papachristodoulou, Pär-Daniel Sundvall

**Affiliations:** a Medpro Clinic Noltorp, Alingsås, Sweden; b Research, Education, Development & Innovation, Primary, Health Care, Region Västra Götaland, Gothenburg, Sweden; c School of Public Health and Community Medicine, General Practice/Family Medicine, Institute of Medicine, Sahlgrenska Academy, University of Gothenburg, Gothenburg, Sweden; d Department of General Practice, The Antibiotic Centre for Primary Care, Institute of Health and Society, University of Oslo, Oslo, Norway; e Department of General Practice, General Practice Research Unit (AFE), Institute of Health and Society, University of Oslo, Oslo, Norway; f Department of Public Health and Clinical Medicine, Family Medicine, Umeå University, Umeå, Sweden; g Kärna Primary Healthcare Center, Region Östergötland, Linköping, Sweden; h Department of Health, Medicine and Caring Sciences, Linköping University, Linköping, Sweden; i Sweden Health Centre Verkstaden, Arvika, Sweden; j Department of Health and Rehabilitation, Institute of Neuroscience and Physiology, Sahlgrenska Academy, University of Gothenburg, Gothenburg, Sweden; k Health Centre Verkstaden, Arvika, Sweden; l Centre for Antibiotic Resistance Research (CARe) at University of Gothenburg, Gothenburg, Sweden

**Keywords:** Acute otitis media, AOM, general practice, primary care, diagnostic methods

## Abstract

**Background:**

Otomicroscopy and pneumatic methods are superior to otoscopy alone in diagnosing acute otitis media (AOM). There is a lack of knowledge regarding the use of different diagnostic methods for AOM in primary health care in Sweden and Norway.

**Methods:**

This cross-sectional study included a questionnaire completed by general practitioners (GPs) and specialist trainees (STs/residents/registrars) working in primary care in Sweden and Norway. Multivariable binary logistic regressions were performed to evaluate the use of diagnostic methods and written advice adjusted for educational level, sex and country.

**Results:**

Otoscopy was the most frequently used method. Sweden had greater access to the more accurate diagnostic methods. In Norway, the following methods were used to a lesser extent: pneumatic otoscopy, adjusted OR 0.15 (95% CI 0.10–0.23; *p* < .001), otomicroscopy, adjusted OR 0.013 (95% CI 0.070–0.027; *p* < .001), pneumatic otomicroscopy, adjusted OR 0.028 (95% CI 0.010–0.078; *p* < .001) and tympanometry, adjusted OR 0.31 (95% CI 0.21–0.45; *p* < .001). Written advice was used to a greater extent in Norway, adjusted OR 4.5 (95% CI 3.1–6.7; *p* < .001). The STs used pneumatic otoscopy and pneumatic otomicroscopy to a lesser extent, adjusted OR 0.65 (95% CI 0.45–0.93; *p* = .019) and 0.63 (95% CI 0.43–0.92; *p* = .016).

**Conclusions:**

Swedish physicians both used and had greater access to the significantly better diagnostic methods compared with Norwegian physicians while the opposite applied to the use of written information. The GPs used pneumatic otoscopy and pneumatic otomicroscopy to a greater extent than STs. Compared with 2012, the Swedish physicians now more frequently used pneumatic otoscopy.

## Introduction

Acute otitis media (AOM) is a frequent infection among children, characterized by a rapid onset of signs and symptoms; middle ear effusion associated with bulging of the tympanic membrane (TM) and pus in the middle ear [1]. The majority of patients with AOM are diagnosed in primary care, and it is one of the most common reasons for children seeking primary care where approximately 80% will experience an AOM during their lifetime [2,3]. Serious complications are rare in high-income countries, in otherwise healthy children, yet include loss of hearing, mastoiditis and meningitis [3,4].

Inspection of the TM is essential to the diagnostics of the ear. Otoscopy is most commonly used; however, relying solely on this as a diagnostic method has limitations in terms of sensitivity and specificity for AOM [5]. Otomicroscopy, on the other hand, has a higher sensitivity and specificity for AOM compared with otoscopy and offers a superior visualization with a binocular view, allowing a detailed assessment of the TM, and enables cleaning of the external acoustic meatus [5–8]. Furthermore, pneumatic otoscopy increases the sensitivity and specificity compared with otoscopy alone. With the use of pneumatic otoscopy/otomicroscopy, it is possible to assess the mobility of the TM and evaluate occurrence of fluid in the middle ear [9,10]. These pneumatic methods increase diagnostic accuracy; although, these methods require further clinical practice by the physician. However, octoscopy, including pneumatic otoscopy, is often preferred to otomicroscopy due to cost and accessibility [2,11]. Previous studies have shown that the most common reason for ignoring certain diagnostic methods is a lack of training and absence of the diagnostic equipment [11–14].

If pneumatic otoscopy or otomicroscopy is unavailable or not fully mastered, tympanometry can, as an addition, facilitate the diagnostics of TM mobility. It requires less practice than pneumatic otoscopy and is a simple method for assessing mobility of the TM [15]. Despite similar sensitivity, it is not possible to discriminate between pus and non-purulent fluid in the middle ear and the diagnosis of AOM cannot be determined solely by the use of tympanometry; hence, it has lower specificity compared with pneumatic otoscopy [2,9]. A combination of otoscopy and tympanometry thus increases the diagnostic certainty regarding whether there is fluid in the middle ear [9,15].

Recommendations regarding the management of AOM differ between countries [16]. The Swedish Medical Products Agency updated the recommendations for diagnostics, treatment and follow-up of AOM in 2010. The more accurate diagnostic methods, pneumatic otoscopy and/or pneumatic otomicroscopy were advocated. Tympanometry was recommended as a supplement to quickly determine the presence of pus/fluid in the middle ear [17]. In Norway, the national recommendations for the diagnostics and treatment of AOM in primary care advocate pneumatic otoscopy [18].

Diagnosing AOM according to guidelines strengthens the probability of a correct diagnosis with increased patient safety with both less over- and underdiagnosis as a result [19]. As AOM tends to be over-diagnosed and over-treated, it is necessary to use correct diagnostic methods in order to avoid unnecessary use of antibiotics [20]. In about 80% of children, an AOM resolves within three days without antibiotic treatment [16,21]. Hence, the Swedish Strategic Programme against antibiotic resistance (Strama) has in recent years actively worked to increase the use of the better diagnostic methods and to distribute written patient information to a greater extent to parents of children with AOM [22].

The aim of this study was to investigate the access to, and use of different diagnostic methods for suspected AOM in Sweden and Norway primary care, as well as the use of written patient information about AOM. The secondary aim was to compare the current use with a previous Swedish study from 2012 [8], and to evaluate if the use of different diagnostic methods and written patient information has changed in Swedish primary care.

## Materials and methods

### Study design and selection

This study used a cross-sectional design and included general practitioners (GPs) and specialist trainees in primary care (STs/residents/registrars) working in primary care centers in Sweden (Region Västra Götaland, Halland, Östergötland, and Västerbotten, Gothenburg, Sweden) and in Norway (all regions). All GPs and STs at the educational meetings and health care centers, where the questionnaires were distributed, were invited to participate. The questionnaires were distributed during 2022/2023. Exclusion criteria were: (1) medical interns and locum/substitute physicians not working as GPs or STs and (2) those who had previously completed the questionnaire at another event or medical meeting in 2022/2023.

### Data collection

Participants were asked to anonymously complete a questionnaire regarding demographic data and diagnostic approach in the management of AOM. The physicians completed the questionnaire in connection with continuing professional education events or medical staff meetings at primary care centers. Drop-outs were considered in order to calculate the response rate.

The project was coordinated at the Research, Education, Development & Innovation, Primary Health Care, Region Västra Götaland, Gothenburg, Sweden where the statistical analyses were performed. The Norwegian part of the study was administered by the Antibiotic Centre for Primary Care at the University of Oslo which was responsible for ethical review in Norway. A similar study was conducted in Region Västra Götaland in 2012 [8] and was compared with the data collected in Region Västra Götaland in the current study.

### Measurements

The questionnaire asked whether the respondent was a GP or ST, years of work experience, employment country (Sweden or Norway) and region (if in Sweden) they worked in, age, sex and the number of patients with AOM they usually managed in a normal month. Furthermore, the use of various diagnostic methods for AOM was considered: otoscopy, pneumatic otoscopy, otomicroscopy, pneumatic otomicroscopy and tympanometry. Five response options were included ranging from ‘always’, ‘often’, ‘sometimes’, ‘seldom’ or ‘never’. The same options were used for the use of oral/written patient information about AOM. Appendix 1 (Supplementary material) includes the questionnaire.

### Statistical analysis

Descriptive statistics were used for demographic data and the use of diagnostic methods.

To evaluate the difference in education level (GP or ST), sex and countries regarding reported use of the different diagnostic methods and oral/written patient information, multivariable binary logistic regressions were performed. Dependent variables were diagnostic methods (otoscopy, pneumatic otoscopy, otomicroscopy, pneumatic otomicroscopy and tympanometry) and use of oral or written patient information dichotomized into ‘never’ (0) or ‘seldom to always’ (1). Independent variables were level of education, sex and country.

To compare the use of different diagnostic methods in Region Västra Götaland, Gothenburg, Sweden between 2012 and 2022/2023, multivariable binary logistic regressions were used. Independent variables were level of education (ST/GP), sex and whether the survey was answered in 2012 or 2022/2023. Analyses were made in IBM SPSS Statistics version 29.0.0.0 (Armonk, NY). The significance level was set at *p* < .05.

### Ethical considerations

The Swedish Ethical Review Authority reviewed this project (31 August 2022, reference number 2022-04112-01) and had no ethical objections. The project did not need an evaluation by the Regional Ethics Committee in Norway (reference number 545257).

## Results

### Group characteristics

A total number of 754 physicians responded to the questionnaire in 2022/2023, 508 (67%) in Sweden and 246 (33%) in Norway. Response rate was 91% (556 distributed questionnaires) in Sweden and 71% (346 distributed questionnaires) in Norway. Demographic data can be found in Table 1.

**Table 1.** Demographic data and access of different diagnostic methods.

	Sweden (*n* = 508)	Norway (*n* = 246)	Total (*n* = 754)
Participants per country % (*n*)	67% (508/754)	33% (246/754)	100% (754/754)
Region in Sweden % (*n*)			
Västra Götaland	37% (187/504)	N/A	
Halland	18% (90/504)	N/A	
Östergötland	38% (194/504)	N/A	
Västerbotten	7% (33/504)	N/A	
Sex female % (*n*)	59% (291/496)	70% (170/244)	62% (461/740)
Age in years, mean (SD)	41.5 (10.2)	40.6 (10.1)	
Clinical role			
GP	42% (196/463)	32% (61/190)	39% (257/653)
ST	58% (267/463)	68% (129/190)	61% (396/653)
Work experience, years, mean (SD)	6.5 (7.5)	7.4 (9.1)	
Number of assessed children with AOM per month % (*n*)			
<5	72% (358/496)	55% (134/242)	67% (492/738)
5–15	26% (127/496)	42% (102/242)	31% (229/738)
>15	2% (11/496)	3% (6/242)	2% (17/738)
Access to % (*n*)			
Otoscopy	99.8% (507/508)	100% (244/244)	99.9% (751/752)
Pneumatic otoscopy	76% (384/504)	24% (56/238)	59% (440/742)
Otomicroscopy	96% (482/502)	3.8% (9/239)	66% (491/741)
Pneumatic otomicroscopy	52% (257/498)	3.3% (8/239)	36% (265/737)
Tympanometry	69% (344/496)	23% (55/239)	54% (399/735)
Written advice	19% (92/496)	51% (120/234)	29% (212/730)

GP: general practitioner; ST: specialist trainee in primary care.

Frequency is described as % of the total number of participants who answered that certain item (*n*).

### Access to diagnostic methods

The proportion of physicians with access to the specific methods were in Sweden and Norway, respectively: pneumatic otoscopy 76% vs. 24%, otomicroscopy 96% vs. 3.8%, pneumatic otomicroscopy 52% vs. 3.3% and tympanometry 69% vs. 23% (Table 1). In Sweden, 19% had access to written advice compared with 51% in Norway (Table 1).

### Use of different diagnostic methods and written advice

The majority (96%) of the physicians always used otoscopy. In total, 83% always gave oral advice, while only 2.8% always provided written advice. Table 2 shows the use of diagnostic methods in Sweden and Norway.

**Table 2.** The use of diagnostic methods for acute otitis media in primary care.

	Never	Seldom	Sometimes	Often	Always
Otoscopy % (*n*)					
Sweden	0.59% (3/505)	1.2% (6/505)	0.39% (2/505)	2.8% (14/505)	95% (480/505)
Norway	0% (0/244)	0% (0/244)	0% (0/244)	3.3% (8/244)	97% (236/244)
Total	0.40% (3/749)	0.80% (6/749)	0.27% (2/749)	2.9% (22/749)	96% (716/749)
Pneumatic otoscopy % (*n*)					
Sweden	28% (140/500)	26% (130/500)	22% (110/500)	18% (88/500)	6.4% (32/500)
Norway	73% (173/237)	13% (30/237)	8.4% (20/237)	3.0% (7/237)	3.0% (7/237)
Total	42% (313/737)	22% (160/737)	18% (130/737)	13% (95/737)	5.3% (39/737)
Otomicroscopy % (*n*)					
Sweden	20% (96/493)	37% (180/493)	34% (166/493)	9.1% (45/493)	1.2% (6/493)
Norway	95% (223/235)	3.0% (7/235)	0.85% (2/235)	0.42% (1/235)	0.85 % (2/235)
Total	44% (319/728)	26% (187/728)	23% (168/728)	6.3% (46/728)	1.1% (8/728)
Pneumatic otomicroscopy % (*n*)					
Sweden	55% (267/489)	29% (140/489)	12% (60/489)	3.9% (19/489)	0.61% (3/489)
Norway	97% (225/233)	1.7% (4/233)	1.7% (4/233)	0% (0/233)	0% (0/233)
Total	68% (492/722)	20% (144/722)	8.9% (64/722)	2.6% (19/722)	0.42% (3/722)
Tympanometry					
Sweden	47% (230/490)	22% (110/490)	21% (102/490)	8.8% (43/490)	1.0% (5/490)
Norway	76% (182/239)	8.4% (20/239)	8.8% (21/239)	6.3% (15/239)	0.42% (1/239)
Total	57% (412/729)	18% (130/729)	17% (123/729)	8.0% (58/729)	0.82% (6/729)
Oral advice					
Sweden	0.40% (2/504)	0.40% (2/504)	2.0% (10/504)	15% (76/504)	82% (414/504)
Norway	0.41% (1/245)	0.41% (1/245)	2.4% (6/245)	13% (31/245)	84% (206/245)
Total	0.40% (3/749)	0.40% (3/749)	2.1% (16/749)	14% (107/749)	83% (620/749)
Written advice					
Sweden	58% (292/505)	24% (122/505)	12% (61/505)	4.4% (22/505)	1.6% (8/505)
Norway	23% (56/245)	22% (55/245)	28% (68/245)	22% (53/245)	5.3% (13/245)
Total	46% (348/750)	24% (177/750)	17% (129/750)	10% (75/750)	2.8% (21/750)

Frequency is described as % of the total number of participants who answered that certain item (*n*).

There was a difference in the use of pneumatic otoscopy, otomicroscopy, pneumatic otomicroscopy and tympanometry where Swedish physicians used these methods to a greater extent (*p* < .001). Written advice (seldom-always) was more frequent in Norway compared with Sweden, adjusted OR 4.5 (95% CI 3.1–6.7; *p* < .001). The STs used pneumatic otoscopy and pneumatic otomicroscopy to a lesser extent than GPs, adjusted OR 0.65 (95% CI 0.45–0.93; *p* = .019) and adjusted OR 0.63 (95% CI 0.43–0.92; *p* = .016). There were no sex differences (Table 3).

**Table 3.** Use of diagnostic methods and written advice for acute otitis media in children.

	Unadjusted odds ratio (95% CI; *p* value)	Adjusted odds ratio (95% CI; *p* value)
Pneumatic otoscopy		
ST (GP is reference)	0.58 (0.42–0.81; *p* = .001)	0.65 (0.45–0.93; *p* = .019)
Being male	1.4 (0.99–1.8; *p* = .054)	1.1 (0.75–1.6; *p* = .65)
Working in Norway	0.14 (0.10–0.20; *p* ≤ .001)	0.15 (0.10–0.23; *p* ≤ .001)
Otomicroscopy		
ST (GP is reference)	0.63 (0.45–0.87; *p* = .006)	0.75 (0.48–1.2; *p* = .23)
Being male	1.6 (1.2–2.2; *p* = .002)	1.2 (0.77–1.9; *p* = .38)
Working in Norway	0.013 (0.070–0.024; *p* ≤ .001)	0.013 (0.070–0.027; *p* ≤ .001)
Pneumatic otomicroscopy		
ST (GP is reference)	0.58 (0.41–0.81; *p* = .001)	0.63 (0.43–0.92; *p* = .016)
Being male	1.4 (1.0–2.0; *p* = .027)	1.1 (0.78–1.7; *p* = .51)
Working in Norway	0.043 (0.021–0.088; *p* ≤ .001)	0.028 (0.010–0.078; *p* ≤ .001)
Tympanometry		
ST (GP is reference)	0.69 (0.50–0.94; *p* = .021)	0.73 (0.52–1.0; *p* = .063)
Being male	1.3 (0.95–1.8; *p* = .097)	1.0 (0.75–1.5; *p* = .79)
Working in Norway	0.28 (0.20–0.39; *p* ≤ .001)	0.31 (0.21–0.45; *p* ≤ .001)
Written advice		
ST (GP is reference)	0.88 (0.64–1.2; *p* = .41)	0.75 (0.54–1.1; *p* = .094)
Being male	0.69 (0.51–0.93; *p* = .015)	0.87 (0.62–1.2; *p* = .43)
Working in Norway	4.6 (3.3–6.5; *p* ≤ .001)	4.5 (3.1–6.7; *p* ≤ .001)

GP: general practitioner; ST: specialist trainees in primary care.

### Comparison with the study from 2012

When comparing the Swedish study from 2012(8) in Region Västra Götaland, pneumatic otoscopy was used to a greater extent in 2022/2023, adjusted OR 2.2 (95% CI 1.1–4.1; *p* = .018) (Table 4).

**Table 4.** Comparison of the use of diagnostic methods and written advice in 2012 and 2022/2023 in Region Västra Götaland, Gothenburg, Sweden.

	Adjusted odds ratio (95% CI; *p* value)
Pneumatic otoscopy	
ST (GP is reference)	0.38 (0.19–0.74; *p* = .005)
Being male	0.83 (0.46–1.5; *p* = .52)
Year 2022 (2012 is reference)	2.2 (1.1–4.1; *p* = .018)
Otomicroscopy	
ST (GP is reference)	0.22 (0.10–0.48; *p* ≤ .001)
Being male	0.85 (0.45–1.6; *p* = .59)
Year 2022 (2012 is reference)	0.82 (0.40–1.7; *p* = .59)
Tympanometry	
ST (GP is reference)	0.60 (0.34–1.0; *p* = .064)
Being male	0.74 (0.45–1.2; *p* = .25)
Year 2022 (2012 is reference)	0.80 (0.46–1.4; *p* = .43)
Written advice never/seldom-always	
ST (GP is reference)	0.86 (0.50–1.5; *p* = .57)
Being male	0.79 (0.48–1.3; *p* = .36)
Year 2022 (2012 is reference)	0.70 (0.41–1.2; *p* = .2)

GP: general practitioner; ST: specialist trainees in primary care.

## Discussion

The superior diagnostic methods pneumatic otoscopy, otomicroscopy, pneumatic otomicroscopy and tympanometry were more accessible and used to a greater extent in Sweden compared with Norway, and the opposite applied for written advice. The GPs used pneumatic otoscopy and pneumatic otomicroscopy to a greater extent than STs. Compared with 2012, the Swedish physicians now used pneumatic otoscopy more frequently.

The lack of access to more advanced equipment might be related to negative attitudes about the technique. If unexperienced with a certain technique, there is lower probability of purchasing the equipment [10]. A likely explanation for the lack of otomicroscopy is the cost. However, the use and access of the pneumatic otoscope were low in Norway, although it is an inexpensive method and advocated by the Norwegian guideline [18]. Furthermore, organizational differences between the two countries could affect the ability to purchase expensive equipment.

The GPs used the diagnostic method pneumatic otoscopy and pneumatic otomicroscopy more often than STs. This illustrates the importance of teaching practical skills of the diagnostic methods in the specialist training program for general practice. The teaching of pneumatic otoscopy is dependent on the physicians. A previous study concluded that a multimodal, interactive workshop increased confidence in diagnosis of AOM and for using pneumatic otoscopy and tympanometry [14]. The use of pneumatic otoscopy should be emphasized during medical training and the GPs need to practice these techniques in order to implement them in their practices [23]. Furthermore, training STs in pneumatic otoscopy can be enhanced with video recordings of the TM [14]. Thus, increased focus on the importance of diagnostics in otitis in the specialist training and antimicrobial stewardship efforts is suggested.

Physicians in the present study used pneumatic otoscopy more frequently compared with the previous Swedish study from 2012. This may be a result of Strama’s efforts to promote and increase the use of the better diagnostic methods when suspecting AOM [22].

## Clinical implications

The results provide knowledge about current access to, and use of, diagnostic methods and patient information for AOM in Swedish and Norwegian primary care, and the change in Sweden since 2012. The more accurate methods including pneumatic otoscopy, otomicroscopy, pneumatic otomicroscopy and tympanometry ought to be used to a greater extent than found in this study. This knowledge is important for the design of clinical guidelines and antibiotic stewardship interventions. It further shows the importance of efforts to increase access and use of the diagnostic methods in primary health care centers. The specialist trainee program should include hands-on training in pneumatic otoscopy and pneumatic otomicroscopy as STs perform these examinations to a lesser extent than GPs. The results found in this study can be of help in the planning of continued educational efforts in Sweden and Norway.

## Strengths and limitations

There are several strengths of this study, including a large number of physicians from two countries. Furthermore, it includes a 10-year follow-up from a previous study, evaluating change in the use of diagnostic methods. The inclusion of both GPs and STs enables comparison between levels of professional experience. However, a weakness of the study was the distribution of GPs and STs, where 61% accounted for STs. This was because the majority of the participants at the educational meetings where the survey was distributed were STs. The inherent limitation of a self-reported questionnaire could imply a weakness, as there is a risk of both recall and response bias. However, the questionnaire was filled out anonymously, administered to several educational meetings including physicians from a variety of centers and the questionnaire was designed to limit potential biases.

## Conclusions

Swedish physicians both used, and had greater access to the significantly better diagnostic methods compared with Norwegian physicians while the opposite applied to the use of written information. The GPs used pneumatic otoscopy and pneumatic otomicroscopy to a greater extent than STs. Compared with 2012, the Swedish physicians now more frequently used pneumatic otoscopy.

## Supplementary Material

Supplemental Material
