# Supplementary material for: Diagnostic methods and written advice for acute otitis media in primary health care
Source: Scand J Prim Health Care. 2024 May 15;42(4):532–7. doi: 10.1080/02813432.2024.2352444 (PMC11552276; doi:10.1080/02813432.2024.2352444)
Supplement: Supplemental Material [file IPRI_A_2352444_SM3664.doc]

**Questionnaire regarding diagnostic methods and written/oral information of acute otitis media (AOM) in children**

***Please answer by circling/ticking the most suitable option for you. You can only participate once. The questionnaire is answered anonymously (ie, do not write your name).***

| 1. Profession: | | General Practitioner | | | | Specialist trainee in primary care | | |
| --- | --- | --- | --- | --- | --- | --- | --- | --- |
| 2. How many years have you worked as a general practitioner/specialist trainee in primary care | | ______ years Your age: ______ years | | | | | | |
| 3. In which region do you work | | Västra Götaland Halland  Östergötland Västerbotten | | | | | | |
| 4. Are you? | | Female | | | | Male | | |
| **5. How many children with AOM do you see per month?** | | <5 children  5-15 children  >15 children | | | | | | |
| **6. What diagnostic methods do you use to diagnose AOM in children?** | | | | | | | | |
| Otoscopy | Always | | | Often | Sometimes | | Seldom | Never |
| Pneumatic otoscopy | Always | | | Often | Sometimes | | Seldom | Never |
| Otomicroscopy | Always | | | Often | Sometimes | | Seldom | Never |
| Pneumatic otomicroscopy | Always | | | Often | Sometimes | | Seldom | Never |
| Tympanometry (as complement to the above) | Always | | | Often | Sometimes | | Seldom | Never |
| **7. Do you have access to the following methods at your primary health care centre?** | | | | | | | | |
| |  | No | Yes | Yes, including in rooms specified for infectious diseases | Do not know | | --- | --- | --- | --- | --- | | Otoscopy |  |  |  |  | | Pneumatic otoscopy |  |  |  |  | | Otomicroscopy |  |  |  |  | | Pneumatic otomicroscopy |  |  |  |  | | Tympanometry |  |  |  |  | | Printed written information about AOM |  |  |  |  | | | | | | | | | |
| **8. How often do you provide parents with oral information regarding follow-up and pain relief for children with AOM?**  **9. How often do you provide parents with written information regarding AOM?** | | | | Oral information: | | | | | | | --- | --- | --- | --- | --- | --- | | Always | Often | Sometimes | Seldom | | Never | |  |  |  |  |  | | | Written information: | | | | | | | Always | Often | Sometimes | Seldom | | Never | | | | | | |
